# Intelligent fault diagnosis and operation condition monitoring of transformer based on multi-source data fusion and mining

**DOI:** 10.1038/s41598-025-91862-8

**Published:** 2025-03-04

**Authors:** Jingping Cui, Wei Kuang, Kai Geng, Pihua Jiao

**Affiliations:** 1 Ural International Institute of Rail Transit, Shandong Polytechnic, Jinan, 250104 China; 2 Jinan Zhongran Technology Development Co., Ltd, Jinan, 250104 China; 3 Shandong Huineng Electric Co., Ltd, Zibo, 255022 China; 4 School of Mechanical and Electronic Engineering, Shandong Agriculture and Engineering University, Zibo, 255300 China

**Keywords:** Energy grids and networks, Power distribution, Power stations

## Abstract

Transformers are important equipment in the power system and their reliable and safe operation is an important guarantee for the high-efficiency operation of the power system. In order to achieve the prognostics and health management of the transformer, a novel intelligent fault diagnosis of the transformer based on multi-source data fusion and correlation analysis is proposed. Firstly, data fusion for multiple components of transformer dissolved gases is performed by an improved entropy weighting method. Then, the combination of bidirectional long short-term memory network, attention mechanism, and convolution neural network is employed to predict the load rate, upper oil temperature, winding temperature data, and the fusion indices of dissolved gas components in the transformer. Furthermore, Apriori correlation analysis is performed on the transformer load rate and upper oil layer, winding temperature, and fusion indices of gas components by support and confidence levels to achieve a predictive assessment of the transformer state. Finally, the validity of the algorithm is verified by applying actual data from a power system monitoring platform. The results show that in the vicinity of sample point 88, the dissolved gas, upper oil temperature, and winding temperature data are not within the normal range of intervals, and it is presumed that the arc discharge phenomenon. Furthermore, the average correct fault diagnosis rate of 100 diagnoses of the transformer fault diagnosis model proposed in this paper is 0.917, and the mean square error of the correct rate is 0.018. The proposed model can achieve the prediction of the accident early warning, to prevent further expansion of the accident.

## Introduction

Transformers are widely used in power systems and are the most important equipment in power supply and distribution, assuming the role of electrical energy supply in residential electricity, industrial parks, and public services^1,2^. Irregularities such as poor contact of the transformer tap changer, short circuits of winding turn-to-turn, blockage of oil passages, and cooling system failures can lead to changes in the composition of dissolved gases, oil temperature, and winding temperature of the internal transformer^3–5^. The traditional method of transformer governance and condition assessment is mainly based on the analysis of the problem of status quo and real-time assessment, observing the transformer operating parameters through the monitoring platform and setting alarm thresholds^6,7^. Maintenance personnel can take appropriate measures after receiving the alarm for sudden abnormal events without warning. Inversely, for the relatively gentle changes in the working conditions, the performance of the early warning ability is insufficient^8,9^. Therefore, how to effectively carry out the short-term state predictive assessment of transformers realize the prediction and early warning, and take preventive measures to avoid the occurrence of faults is a key issue in the construction of a strong smart grid^10^.

There are some conventional approaches to predict the power transformer data, such as the autoregressive integral moving average (ARIMA)^11,12^, random walk (RW)^13^, generalized autoregressive conditional heteroscedasticity (GARCH)^14,15^ and vectorial autoregression (VAR)^16^. These conventional approaches have satisfactory prediction performance for linear correlation variables, but they cannot capture the nonlinear characteristics of data. Due to the limitations of conventional approaches, plentiful nonlinear artificial intelligence and deep learning methods rise in response to the proper time and conditions^17–20^, and can be employed for data prediction based on the time series, such as artificial neural network (ANN)^21^, support vector machine (SVM)^22^ and recurrent neural network (RNN)^23^. However, aiming at the problems of gradient explosion and disappearance in RNN, the long short-term memory network (LSTM) has been developed based on RNN^24,25^. The effective message can be extracted through the various gate structures of LSTM and gathered in historical data during the training process. In addition, in contrast to LSTM, BiLSTM consists of two layers of LSTM, which can take full advantage of both forward and reverse information^26^. Therefore, it is especially suitable for solving data forecasting problems based on the time series. Furthermore, considering that historical data contribute differently to data at different points in time, while current neural network models (including BiLSTM) contribute equally to each point in time to be predicted, attention mechanisms have been developed^27^. In order to further improve the prediction accuracy of the models, Attention mechanisms are usually introduced in deep learning models. Attention mechanisms can obtain valid information and important spatiotemporal features from new coding sequences. One of the drawbacks of conventional neural networks is the poor scalability due to the complete connectivity of neurons, which is overcome by convolutional neural networks (CNN). CNN enhances the efficiency of the algorithm and decreases the number of parameters. Many kinds of literature have demonstrated that CNN has the advantage of extraction and reorganization^28,29^. Therefore, based on the advantages of extracting effective information from the Attention mechanism layer and CNN capturing the hierarchical structure, it is of great importance to study the combination model to predict the power transformer data.

With the application and development of smart grids, transformers are also gradually developing in the direction of intelligence^30^. Transformer online monitoring can provide real-time monitoring of transformer operation data. Through the processing and analysis of big data, transformer failure can achieve early discovery and early treatment, which is conducive to solving the problem of transformer condition evaluation and prediction^31^. Therefore, judging and evaluating the transformer status based on data prediction and data mining offers a different approach to online monitoring of transformer faults. Multi-source data fusion and data extraction is an advanced data technology for data-driven insights and data correlation analysis, i.e. identifying the relationships, trends, and linkages between massive and complicated datasets^32^. Multiple factors hamper the modeling and analysis of their interaction and complex relationships with the creation of data sources for knowledge acquisition and, eventually, the process of decision finding. Consequently, leveraging the intellectual properties of models that can handle massive and sophisticated datasets can lead to more acceptable results. A wide range of investigations in the last two decades have taken advantage of the possibilities offered by data excavation techniques in diverse fields, such as prediction of atmospheric pollutant levels^33^, prediction of the optical depth of aerosols^34^, and mapping of subsidence susceptibility^35^.

Apriori is a powerful approach based on information retrieval. It has been deployed for application in learning exploration and forecasting, such as wind speed^36^, landslides^37^ and road accidents^38^. Research in the area of smart diagnostics for power devices typically utilizes various methods of machine learning, such as ANN, Support Vector Machines (SVM), and Random Forests (RF), as well as data exploration techniques, such as Boosted Generalized Additive Models (BGAM), which seek to determine correlations through modeling the mathematical relationships among various performance properties^39–42^. In spite of the established performance of these techniques, they are not able to deliver connection patterns between the events and the contributory factors. Thus, the approaches are not universal, and for implementation in other areas, complicated parameters need to be reassigned and re-run. The primary strength of the Apriori approach is that, on the basis of the patterns it produces for an occurrence, it is feasible to extrapolate these patterns to similar occurrences and detect associations without having to re-run the procedure. The Apriori methodology, a powerful rule-based data exploration technique, is deployed for the first time in the current work to detect faults through the detection of association patterns by analyzing the complicated behavior of various factors in the transformer load rate and the top oil layer, winding temperature and fusion indices of the gas components. Hence, concrete decisions can be undertaken to enhance the applicability of transformer fault analysis methods by reducing handling costs, decreasing data demands, and eliminating associated problems.

How to play the role of data mining methods to support the stable operation of transformers based on the means of online monitoring of data, how to realize the intelligent diagnosis of transformer operation status by using the constantly changing operation data, as well as to realize the predictive assessment and warning of transformers are still the outstanding issues of the intelligent operation of transformers. To the best knowledge of the authors of this paper, there are no works of literature on the application of the intelligent fault diagnosis of transformers based on multi-source data fusion and data mining. Given the context discussed, the contribution of this paper is fourfold:

1. Intelligent fault diagnosis of the transformer based on multi-source data fusion and data mining is modeled to realize the stable operation of the transformer under different operating conditions by predictive assessment and early warning.
2. The components of dissolved gas, upper oil temperature, winding temperature, and load rate of the transformer are selected as state characteristic parameters, and data fusion is performed on the multiple components of dissolved gas of the transformer.
3. The state characteristic parameters of the transformer are predicted by CNN-BiLSTM-Attention to ensure the basis of data application and data accuracy in the predictive evaluation of the power transformer.
4. The correlation analyses of dissolved gas composition, upper oil temperature, and winding temperature under different load rates are achieved by multi-source data fusion and Apriori correlation analysis.

The remainder of the paper is organized as follows. Section “Transformer state characteristic parameter selection and data fusion” gives the transformer state characteristic parameter selection and data fusion. Section “Data prediction” deploys the data prediction model based on CNN-BiLSTM-Attention fusion. Section “Intelligent fault diagnosis of transformers based on correlation analysis” further proposes the intelligent fault diagnosis of transformers based on correlation analysis. In “Experimental results” Section, experimental results are investigated to demonstrate the proposed method. Section “Conclusions” draws the main conclusions.

## Transformer state characteristic parameter selection and data fusion

In this paper, the condition assessment of the transformer is proposed to be related to the composition of dissolved gases in the transformer, the upper oil temperature, the winding temperature, and the load rate, where the composition of the transformer oil mainly contains $\hbox {H}_{2}$, $\hbox {CH}_{4}$, $\hbox {C}_{2}\hbox {H}_{2}$, $\hbox {C}_{2}\hbox {H}_{4}$, $\hbox {C}_{2}\hbox {H}_{6}$, $\hbox {CO}$ and $\hbox {CO}_{2}$. The documented basis for fault diagnosis of dissolved gas in transformer oil mainly includes GB/T 7252-2001 Guidelines for Analysis and Judgement of Dissolved Gas in Transformer Oil and other related standards or guidelines. These documents provide the framework of transformer fault diagnosis based on dissolved gas analysis and the corresponding fault judgment basis. The content in the oil mainly includes $\hbox {H}_{2}$, $\hbox {CH}_{4}$, $\hbox {CO}$, $\hbox {CO}_{2}$, $\hbox {C}_{2}\hbox {H}_{6}$, $\hbox {C}_{2}\hbox {H}_{4}$, $\hbox {C}_{2}\hbox {H}_{2}$ and other gases. Different types of faults produce different changes in gas composition and content. Therefore, The data fusion of multiple compositions of transformer dissolved gases, the upper oil temperature, and the winding temperature are used as the characteristic parameters for condition assessment in this paper.

The multiple components of the transformer dissolved gas are first normalized and the *j*-th component $p_{ij}$ on the *t*-th time scale after normalization can be expressed as:

1 $$\begin{aligned} p_{tj}=v_{tj}/\sum _{j=1}^{m}v_{tj} \end{aligned}$$

where $v_{tj}$ denotes the *j*-th component on the *t*-th time scale before normalization.

The entropy value $e_{j}$ of the *j*-th component can be denoted as:

2 $$\begin{aligned} e_j=-K\sum _{j=1}^m\bigl ( p_{ij}\times \ln p_{ij}\bigr ) \end{aligned}$$

where $K=1/ {\textrm{ln}} m$. If $p_{ij}=0$, then $\underset{p_{ij}\rightarrow 0}{\lim } p_{ij}\ln p_{ij}=0$.

Thus, the entropy weights can be expressed as follows.

3 $$\begin{aligned} w_j=1-e_j/\sum _{j=1}^m\left( 1-e_j\right) \end{aligned}$$

The expected value *E* of the weights is obtained by adaptive optimization, which can be defined as:

4 $$\begin{aligned} E=\sum _{j=1}^mw_jv_{tj} \end{aligned}$$

A prediction error exists when the actual value *REA* and the predicted expected value are not equal, then the prediction error can be shown as follows.

5 $$\begin{aligned} ERR=\frac{1}{2}\sum _{j=1}^m\left( REA_j-E_j\right) ^2 \end{aligned}$$

where *ERR* indicates the prediction error between the actual value and the predicted expected value.

The weights are continuously adjusted by using an error gradient descent algorithm, then the adjusted values of weights can be defined as:

6 $$\begin{aligned} \Delta w_j=-\eta \frac{\partial ERR}{\partial w_j} \end{aligned}$$

where the negative sign indicates a gradient decrease and $\eta$ is the scaling factor.

7 $$\begin{aligned} \begin{aligned} \frac{\partial ERR}{\partial w_j}&=\frac{\partial }{\partial w_{j}}\frac{1}{2}\sum _{j=1}^{m}\Big (REA_{j}-E_{j}\Big ) \\&= \frac{1}{2}\frac{\partial }{\partial w_{j}}\sum _{j=1}^{m}\left( REA_{j}-E_{j}\right) ^{2} \\&=\frac{1}{2}\sum _{j=1}^{m}2\Big (REA_{j}-E_{j}\Big )\frac{\partial }{\partial w_{j}}\Big (REA_{j}-E_{j}\Big ) \\&=\sum _{j=1}^m\Big (REA_j-E_j\Big )\frac{\partial }{\partial w_j}\Big (REA_j-w_jz_j\Big ) \\&=\sum _{j=1}^m\left[ \left( REA_j-E_j\right) \times \left( -z_j\right) \right] \end{aligned} \end{aligned}$$

Therefore, based on the adjusted values of weights, the weights can be updated by iteration:

8 $$\begin{aligned} w_j=w_{j-1}+\Delta w \end{aligned}$$

The dissolved gas index on the *t*-th time scale is:

9 $$\begin{aligned} F_t{=}w_1\times \nu _{t1}+w_2\times \nu _{t2}+\cdots w_m\times \nu _{tm} \end{aligned}$$

## Data prediction

**Fig. 1:**
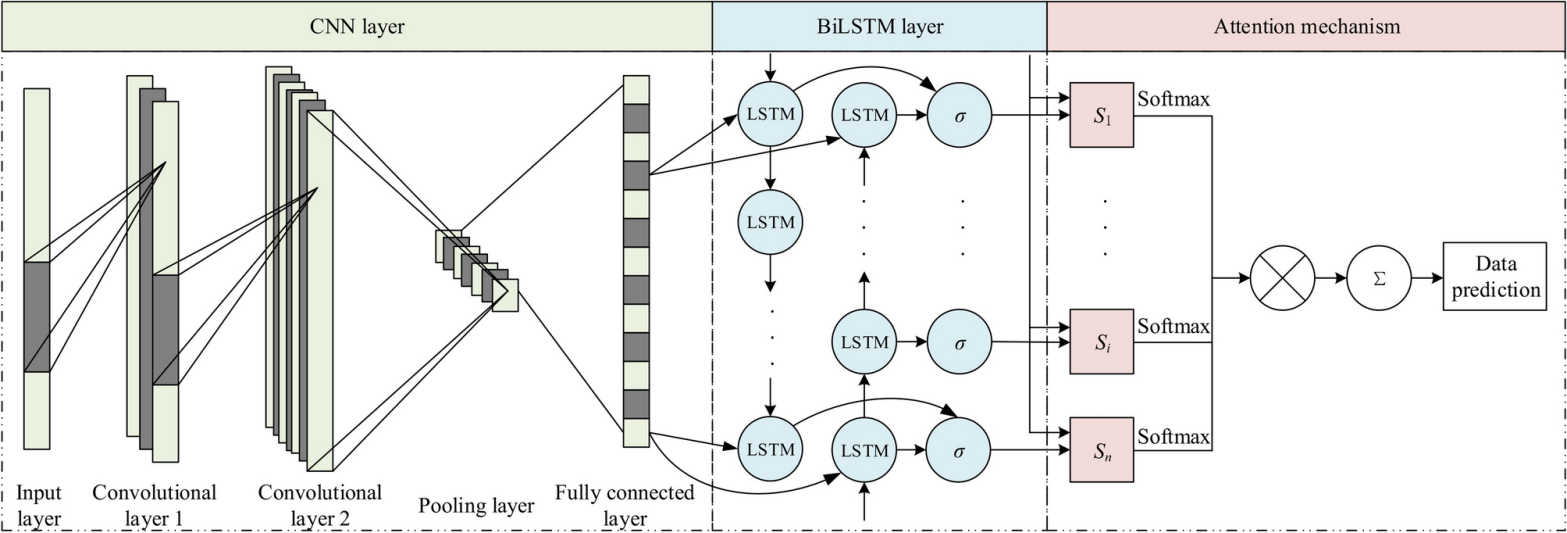
Network structure of CNN, BiLSTM and Attention model.

The network structure of the data prediction model based on CNN-BiLSTM-Attention fusion is shown in Fig. 1. For the input data, firstly, the longitudinal feature extraction module with convolutional layer as the core is passed, and on the basis of this, the horizontal feature extraction module with BiLSTM network as the core is passed. These two modules are cascaded back and forth to fully explore the data features from both longitudinal time points and horizontal time series perspectives. By adding the attention mechanism, the model pays more attention to the feature change pattern of the data near the moment of fault occurrence. Compared with the complex deep learning network structure, the model has a simple structure and faster speed, which can achieve timely and accurate data prediction.

### CNN network

CNN is a deep learning network with a convolutional structure that extracts detailed features of data and is commonly used in the field of image classification. In the longitudinal feature extraction module, each CNN layer includes three computations: convolution, normalization, and activation function. Taking the winding temperature data as input, the formula for the convolution operation can be defined as:

10 $$\begin{aligned} z_j^l=\sum _{i=1}^m z_j^{l-1}*k_{ij}^l+b_j^l,j=1,\ldots,n \end{aligned}$$

where $z_j^l$ represents the feature vector of the *j*-th feature surface of the convolutional output, $z_j^{l-1}$ represents the winding temperature signal of the *i*-th input feature surface, $k_{ij}$ represents the parameter of the *j*-th convolution kernel connected to the *i*-th input feature surface, and $b_j^l$ represents the bias of the *j*-th convolution kernel.

Convolution is able to extract linear features of the data by performing a linear computation of multiplying and summing the elements of the input data with a convolution kernel, while the convolution operation moves over the input sequence by sliding the convolution kernel in a way that captures structural features of local patterns in the input data.

As the model is trained, the distribution of data will be shifted. Therefore, normalization is used to avoid the problem of gradient disappearance caused by the data falling into the saturation region of the activation function and to speed up the convergence of the model. The normalization formula is as follows:

11 $$\begin{aligned} z_{LN}=\frac{z-E(z)}{\sqrt{Var(z)+\epsilon }}\times \alpha +\beta \end{aligned}$$

where *z* is the result of the convolution operation on the winding temperature, *E*(*z*) is the mean, *Var*(*z*) is the variance, $\epsilon$ is a small amount greater than 0 to prevent the denominator from being zero, which is generally taken as $10^{-5}$, $\alpha$ and $\beta$ are trainable parameters.

The essence of the activation function is to perform a nonlinear transformation of the input data to extract the nonlinear features of the data and increase the fitting ability of the network. The activation function used in this paper is the ReLU function:

12 $$\begin{aligned} x=\operatorname {Re}\operatorname {LU}(z_{LN})= {\left\{ \begin{array}{ll} z_{LN}& \quad if \ z_{LN}\ge 0\\ 0& \quad if \ z_{LN}<0 \end{array}\right. } \end{aligned}$$

Compared with other activation functions, the ReLU function solves the problem of vanishing gradient on positive intervals. In addition, since it only needs to judge whether the input is greater than zero, its computational speed and convergence speed are faster, which lays the foundation for the model to be able to achieve data prediction quickly. The feature vectors are passed into the pooling layer, which calculates the average value of the data for each output channel, increasing the robustness of the model and reducing the number of parameters, which prevents model overfitting and speeds up model convergence. The next flattening layer unfolds the data of each convolutional channel in one dimension for the transition between CNN and BiLSTM.

### BiLSTM network

**Fig. 2:**
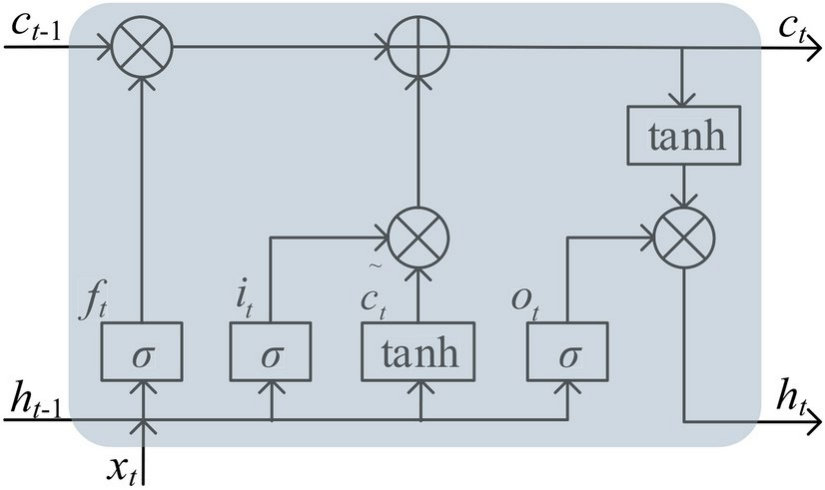
LSTM unit structure.

The BiLSTM network is a variant of the LSTM network and is formed by combining the forward LSTM network and the backward LSTM network. LSTM network is an improvement of Recurrent Neural Network (RNN), which improves the short-term memory problem of RNN due to the disappearance of gradient, which causes the more distant information to have almost no effect on the current moment by adding three special gate structures and memory units. The LSTM unit structure is shown in Fig. 2.

The input set is $\{ x_{1}, x_{2},\ldots, x_{t} \}$, where $x_{t}= \{ x_{t,1}, x_{t,2},\ldots, x_{t,k} \}$, which denotes the *k*-dimensional vector data at time *t*. The forgetting gate $f_{t}$, the candidate state of the memory cell ${\tilde{c}}_{t}$, the input gate $i_{t}$, the state of the memory cell $c_{t}$, the output gate $o_{t}$ and the hidden layer output value $h_{t}$ can be expressed as follows.

13 $$\begin{aligned} \left\{ \begin{array}{l} f_{t}=\sigma \left( W_{f} x_{f}+U_{f} h_{t-1}+b_{f}\right) \\ {\tilde{c}}_{t}=\tanh \left( W_{c} x_{t}+U_{c} h_{t-1}+b_{c}\right) \\ i_{t}=\sigma \left( W_{i} x_{t}+U_{i} h_{t-1}+b_{i}\right) \\ o_{t}=\sigma \left( W_{o} x_{t}+U_{o} h_{t-1}+b_{o}\right) \\ c_{t}=f_{t} \otimes c_{t-1} \oplus i_{t} \otimes {\tilde{c}}_{t} \\ h_{t}=o_{t} \otimes \tanh \left( c_{t}\right) \end{array}\right. \end{aligned}$$

where $W_{f}$ is the weight matrix of the oblivious gate, $b_{f}$ is the bias of the oblivious gate, $\sigma$ and *Tanh* denote the sigmoid and hyperbolic tangent activation function, $W_{c}$, $W_{i}$, $b_{c}$ and $b_{i}$ denote the weight matrix and bias corresponding to the candidate state ${\tilde{c}}_{t}$ and the input gate $i_{t}$, respectively, $W_{o}$ and $b_{o}$ are the weight matrix and bias of the output gate $O_{t}$, $\oplus$ and $\otimes$ denote the add and multiply, respectively.

In addition, the activation functions of $\sigma$ and *Tanh* can be shown as:

14 $$\begin{aligned} \sigma (x)= & 1/\left( 1+\exp (-x)\right) \end{aligned}$$

15 $$\begin{aligned} \textrm{Tanh}(x)= & \left( \exp (x)-\exp (-x)\right) /\left( \exp (x)+\exp (-x)\right) \end{aligned}$$

**Fig. 3:**
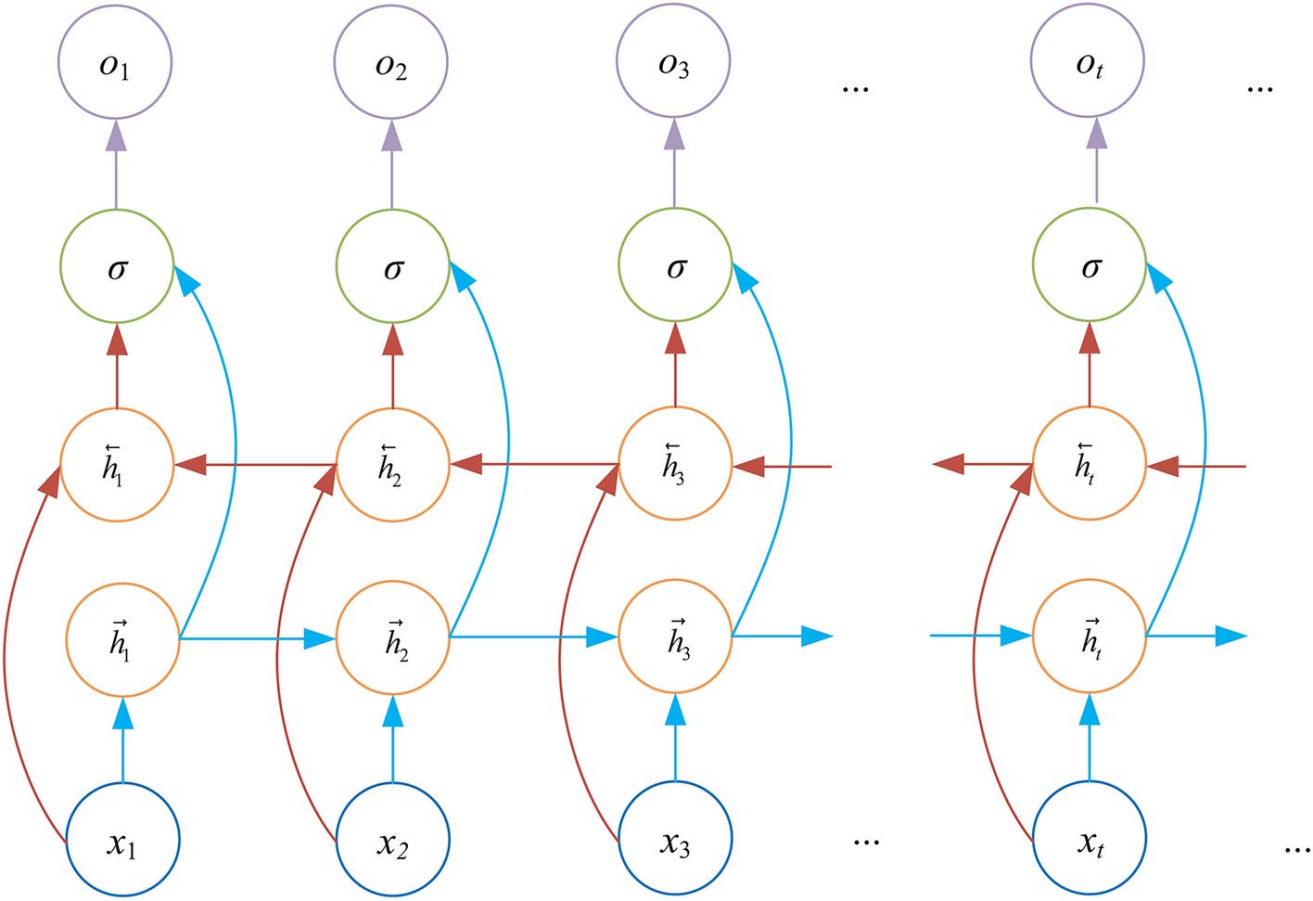
BiLSTM structure.

From the above, it can be seen that the network parameters of LSTM are trained on the data in the order from front to back, which is low utilization of the data and cannot fully extract the intrinsic characteristics of the data in the time series. BiLSTM network combines forward LSTM and backward LSTM, which can simultaneously extract the forward and backward historical transverse features of the data, and further explore the intrinsic connection between the current data and the past and future data, so as to improve the utilization rate of the data and the prediction accuracy of the model. The structure of the BiLSTM network is shown in Fig. 3.

The hidden layer output value $h_{t}$ of BiLSTM consists of forward vector $\overrightarrow{{h}_{t}}$ and inverse vector $\overrightarrow{{h}_{t}}$, where the forward vector and inverse vector output are:

16 $$\begin{aligned} \overrightarrow{{h}_{t}}= & \overrightarrow{\textrm{LSTM}}(h_{t-1},x_{t},{\textbf{c}}_{t-1}),t\in [1,T] \end{aligned}$$

17 $$\begin{aligned} \overleftarrow{{h}_{t}}= & \overleftarrow{\textrm{LSTM}}(h_{t+1},x_{t},{\textbf{c}}_{t+1}),t\in [T,1] \end{aligned}$$

Therefore, the output of BiLSTM at time *t* can be expressed as:

18 $$\begin{aligned} y_t=\sigma (W_y\cdot [\vec {h_t},\overline{h_t}]+b_y) \end{aligned}$$

where $W_y$ and $b_y$ are the weight matrix and bias terms, respectively.

The forward transmission layer extracts the forward history of faults in the direction of the time series, from front to back. The backward transmission layer traces the historical feature correlations of the faults from backward to forward in the reverse direction of the time series. By fusing the two features, the horizontal features of the data are obtained.

### Attention mechanism

**Fig. 4:**
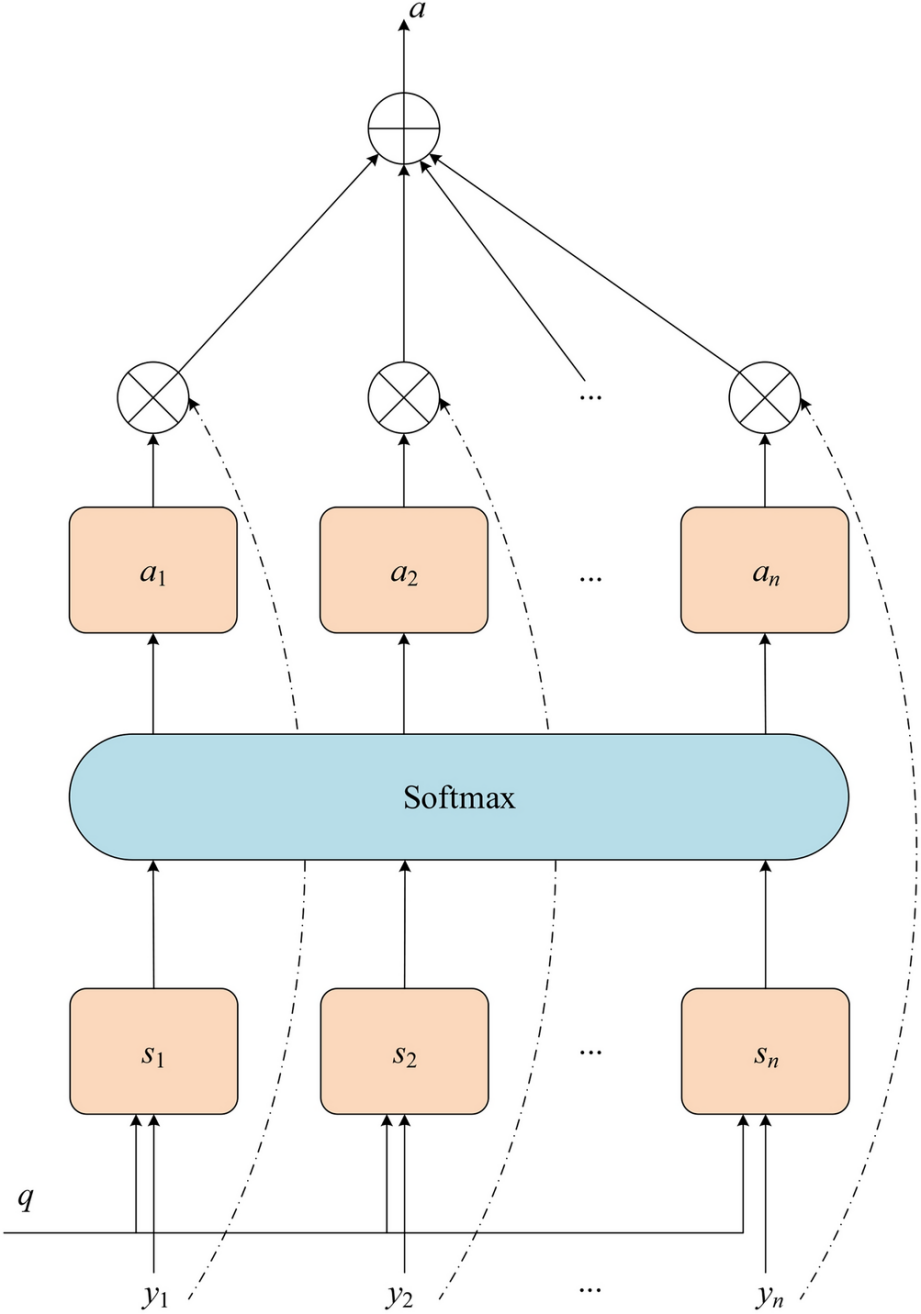
Attention mechanism.

The Attention mechanism is an idea based on human visual attention that assigns different weights to different input features to enhance important features and avoid irrelevant information from influencing the final result, thus improving the performance and effectiveness of the model. The Attention mechanism is depicted in Fig. 4. Specifically, the implementation of an Attention mechanism typically involves the following steps.

*Step 1* A set of query vectors $y=\left[ y_{1}, y_{2},\ldots, y_{n} \right]$, is obtained by encoding the input sequence *q*.

*Step 2* Subsequently, using the scoring function *s*, the expression can be expressed as:

19 $$\begin{aligned} e_{ij}=\tanh (W_1*h_i+W_2*h_j+b) \end{aligned}$$

where $h_i$ and $h_j$ are the hidden layer states, $e_{ij}$ indicates the correlation between the *i*-th state and the *j*-th state, *W* is the weight, *b* represents the offset vector.

*Step 3* Then, the Softmax function is used for normalization to convert the value of each correlation into a probability weigh $a_{i}$, which can be calculated as follows:

20 $$\begin{aligned} a_{ij}=softmax\begin{pmatrix}e_{ij}\end{pmatrix}=\frac{\exp (e_{ij})}{\sum _j\exp (e_{ij})} \end{aligned}$$

where $a_{ij}$ is the attention weight of *j* to *i*, and $\sum _{j} a_{ij}=1$.

*Step 4* The final Attention output value $H_i$ is calculated from the weight coefficients $a_{ij}$ input vector $h_j$ , which is shown as following:

21 $$\begin{aligned} H_i=\sum _j a_{ij}*h_j \end{aligned}$$

## Intelligent fault diagnosis of transformers based on correlation analysis

Based on the load rate of the transformer, the transient stability of the transformer is identified through the multivariate data correlation analysis of the composition of dissolved gas, upper oil temperature, and winding temperature, so as to avoid the situation of leakage and misjudgement of a single data source. Through the correlation analysis of multivariate data, the causal pairs formed by the transformer load rate, the composition of dissolved gases, the temperature of the upper layer of oil, and the temperature of the windings are taken into account, and the correlation rules of support and confidence are used to determine the data ranges corresponding to the correlated data.

The data is divided into *n* intervals based on the maximum and minimum values of the composition of dissolved gases, upper oil temperature, winding temperature, and load rate data of the transformer, which can be expressed as:

22 $$\begin{aligned} {\left\{ \begin{array}{ll} G_\Delta ^\textrm{A}=G_\textrm{max}^\textrm{A}-G_\textrm{min}^\textrm{A}/n\\ O_\Delta ^\textrm{B}=O_\textrm{max}^\textrm{B}-O_\textrm{min}^\textrm{B}/n\\ W_\Delta ^\textrm{C}=W_\textrm{max}^\textrm{C}-W_\textrm{min}^\textrm{C}/n\\ L_\Delta =L_\textrm{max}-L_\textrm{min}/n \end{array}\right. } \end{aligned}$$

where load rate $L= \left( S_{\textrm{real}} / S_{\textrm{rated}} \right) \times 100 \%$, $S_{\textrm{real}}$ represents the apparent power, $S_{\textrm{rated}}$ denotes rated capacity of transformer.

The intervals of the composition of dissolved gases, upper oil temperature, winding temperature, and load rate for the transformer can be shown as follows:

23 $$\begin{aligned} {\left\{ \begin{array}{ll} \left[ G_{\min },G_{\min }+G_{\Delta }\right] ,\left[ G_{\min }+G_{\Delta },G_{\min }+2G_{\Delta }\right] ,\cdots ;\left[ G_{\min }+(n-1)G_{\Delta },G_{\min }+nG_{\Delta }\right] \\ \left[ O_{\min },O_{\min }+O_{\Delta }\right] ,\left[ O_{\min }+O_{\Delta },O_{\min }+2O_{\Delta }\right] ,\cdots ;\left[ O_{\min }+(n-1)O_{\Delta },O_{\min }+nO_{\Delta }\right] \\ \left[ W_{\min },W_{\min }+W_{\Delta }\right] ,\left[ W_{\min }+W_{\Delta },W_{\min }+2W_{\Delta }\right] ,\cdots ;\left[ W_{\min }+(n-1)W_{\Delta },W_{\min }+nW_{\Delta }\right] \\ \left[ L_{\min },L_{\min }+L_{\Delta }\right] ,\left[ L_{\min }+L_{\Delta },L_{\min }+2L_{\Delta }\right] ,\cdots ;\left[ L_{\min }+(n-1)L_{\Delta },L_{\min }+nL_{\Delta }\right] \end{array}\right. } \end{aligned}$$

In order to facilitate the presentation of the causal pairs of data, the composition of the dissolved gases, the upper oil temperature, and the winding temperature intervals may be briefly described as $\{ G_{1}, G_{2},\ldots, G_{n} \}$, $\{ O_{1}, O_{2},\ldots, O_{n} \}$ and $\{ W_{1}, W_{2},\ldots, W_{n} \}$. Similarly, the interval of the load rate of the transformer is abbreviated as $\{ L_{1}, L_{2},\ldots, L_{n} \}$.

In addition, the load rate intervals, the dissolved gases, the upper oil temperature, and the winding temperature intervals can be matched to form causal pairs of load rate and composition of dissolved gas, upper oil temperature, and winding temperature, respectively, which can be expressed as follows.

24 $$\begin{aligned} {\left\{ \begin{array}{ll} \left[ L_1,G_1\right] ,\left[ L_1,G_2\right] ,\ldots,\left[ L_2,G_1\right] ,\left[ L_2,G_2\right] ,\ldots,\left[ L_n,G_n\right] \\ \left[ L_1,O_1\right] ,\left[ L_1,O_2\right] ,\ldots,\left[ L_2,O_1\right] ,\left[ L_2,O_2\right] ,\ldots,\left[ L_n,O_n\right] \\ \left[ L_1,W_1\right] ,\left[ L_1,W_2\right] ,\ldots,\left[ L_2,W_1\right] ,\left[ L_2,W_2\right] ,\ldots,\left[ L_n,W_n\right] \end{array}\right. } \end{aligned}$$

Then, calculate the support level *Sup* and confidence level *Con* of each causal pair:

25 $$\begin{aligned} & {\left\{ \begin{array}{ll} Sup\Big (L_a\cap G_b\Big )=\text {count}\Big (L_a\cap G_b\Big )/N_\text {G}\\ Sup\Big (L_a\cap O_b\Big )=\text {count}\Big (L_a\cap O_b\Big )/N_\text {o}\\ Sup\Big (L_a\cap W_b\Big )=\text {count}\Big (L_a\cap W_b\Big )/N_\text {w} \end{array}\right. } \end{aligned}$$

26 $$\begin{aligned} & {\left\{ \begin{array}{ll} Con\Big (L_a\cap G_b\Big )=\textrm{count}\Big (L_a\cap G_b\Big )/\textrm{count}(L_a)\\ Con\Big (L_a\cap O_b\Big )=\textrm{count}\Big (L_a\cap O_b\Big )/\textrm{count}(L_a)\\ Con\Big (L_a\cap W_b\Big )=\textrm{count}\Big (L_a\cap W_b\Big )/\textrm{count}(L_a) \end{array}\right. } \end{aligned}$$

where $L_{a}$ denotes the interval of $\alpha$-th load rate, $G_b,O_b$ and $W_b$ are the interval of *b*-th dissolved gas upper oil temperature, and winding temperature, respectively, $count\left( L_{a}\cap G_{b}\right)$ represents the number of causal pairs belonging to both the *a*-th load rate interval and the *b*-th interval of the dissolved gas, $N_\textrm{G}$ indicates the total number of causal pairs in the set of load rate and dissolved gases, $$count\begin{pmatrix}L_a\cap O_b\end{pmatrix}$$ represents the number of causal pairs belonging to both the *a*-th load rate interval and the *b*-th interval of the upper oil temperature, $N_\textrm{G}$ indicates the total number of causal pairs in the set of load rate and the upper oil temperature, $count\left( L_{a}\cap W_{b}\right)$ represents the number of causal pairs belonging to both the *a*-th load rate interval and the *b*-th interval of the winding temperature, $N_\textrm{G}$ indicates the total number of causal pairs in the set of load rate and winding temperature.

**Fig. 5:**
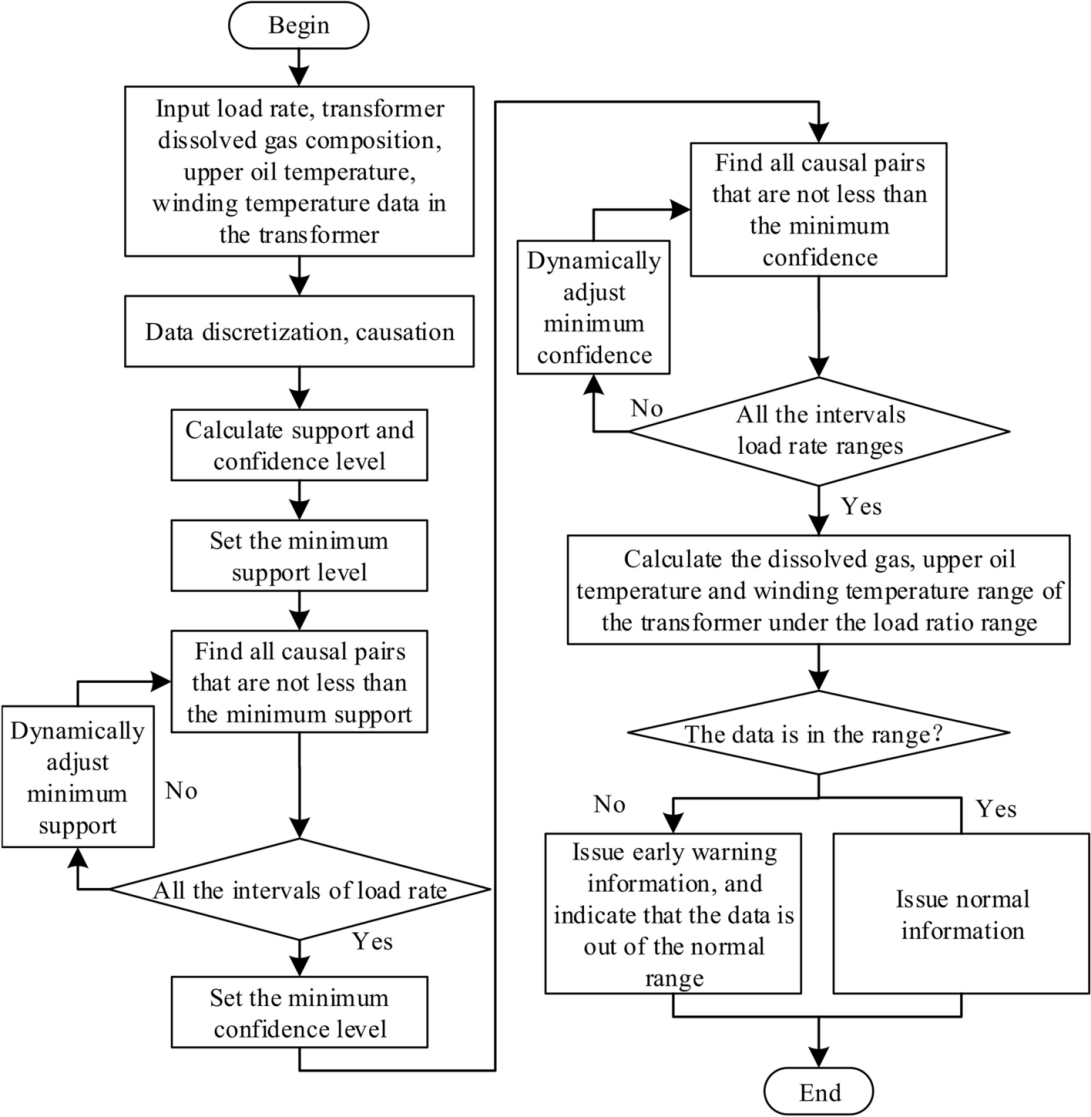
The flowchart for correlation analysis of load rate, dissolved gas, oil temperature of upper layer and winding temperature.

The causal pairs of the dissolved gases in the transformer, the upper oil temperature, and the winding temperature need to be greater than or equal to the threshold of minimum support and confidence at the same time, and the flowchart of correlation analysis for load rate, dissolved gas, oil temperature of upper layer and winding temperature can be shown in Fig. 5.

## Experimental results

**Fig. 6:**
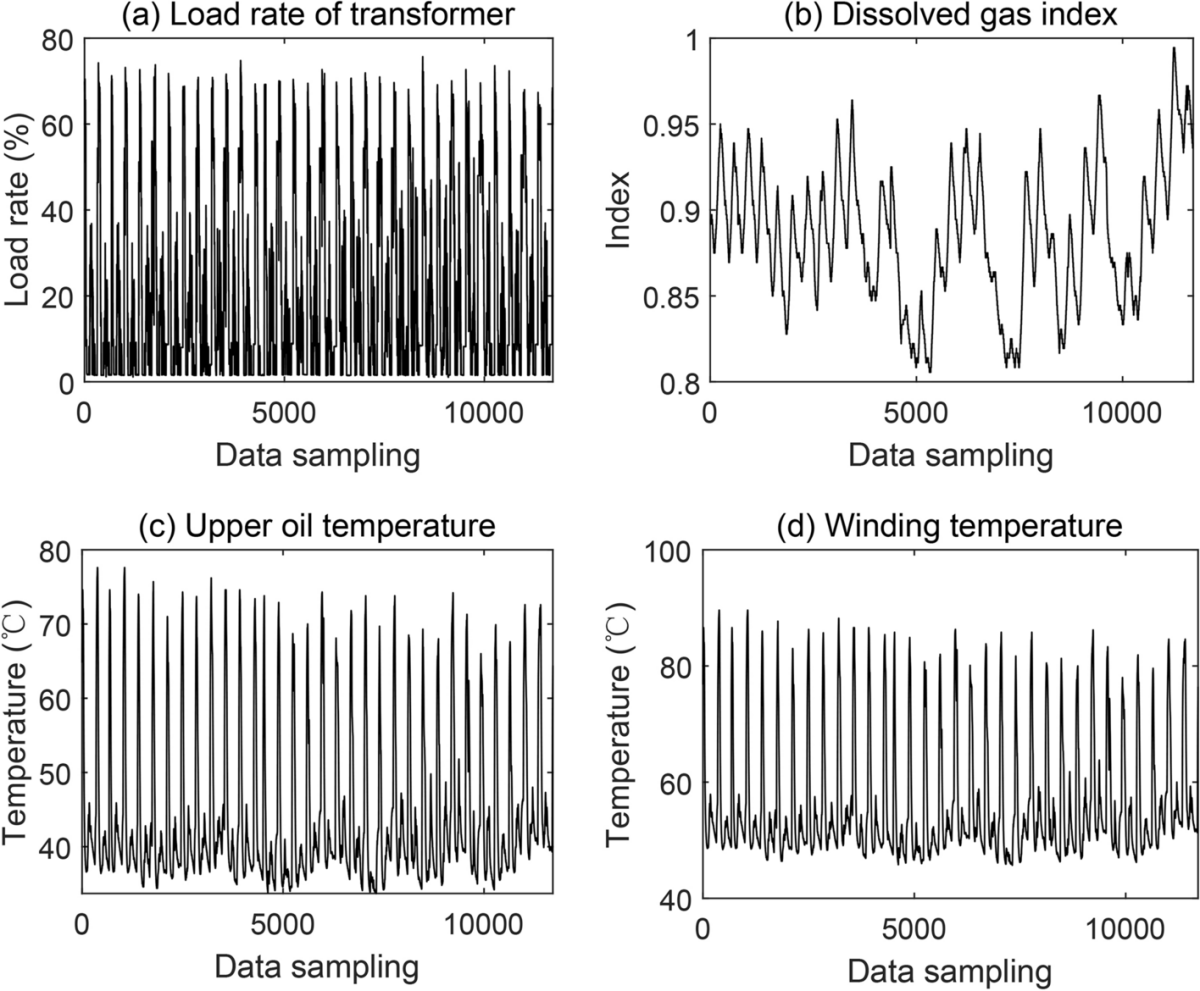
The sample of data sets.

In this paper, a three-phase oil-immersed amorphous alloy distribution transformer was chosen as the object of study in the study of intelligent fault diagnosis of the transformer. The simulation corresponding to the proposed model is implemented in MATLAB platform, on a PC with Intel Core i7, 5.4GHz processor, and 32 GB of memory. The transformer is equipped with a dissolved gas analyzer in oil, and a predictive assessment of transformer condition is performed using the actual transformer load factor, the composition of dissolved gases, upper oil temperature, and winding temperature from a transformer monitoring platform in this paper. The data used are from one sampling point at 15-minute intervals from 1 May 2023 to 31 August 2023 at the substation, and the sample of data sets are shown in Fig. 6. This transformer condition predictive assessment involves multivariate feature inputs and provides a field application example reference for subsequent studies.

The algorithm of the transformer condition predictive assessment method based on correlation analysis is as follows: (1) Data fusion of multiple components of dissolved gases in the transformer is carried out by the improved entropy weight method, and the data after fusion with the dissolved gases, the upper oil temperature, winding temperature, and load rate are selected as the characteristic parameters for condition assessment. (2) Initial training and prediction based on BiLSTM neural network. Select the transformer load rate and the dissolved gas, upper oil temperature, and winding temperature data after data fusion as inputs. Divide the training set and test set, initialize the input dimension, output dimension, iterations, and activation function based on experience, supervise the training of the model according to the gradient descent algorithm, and get the prediction value of the data. (3) Through the correlation analysis of multivariate data, the transformer load rate, the composition of dissolved gas in the transformer, the upper oil temperature, and the winding temperature are considered to form the causal pairs, and the correlation rules of support and confidence are used to determine the data range corresponding to the correlated data. (4) If the predicted dissolved gas, upper oil temperature, and winding temperature are not within the corresponding load ratio interval, the power system monitoring platform will issue a warning signal. If the dissolved gas is not in the corresponding interval, you can check the data in Table 2 to find the corresponding fault information. (5) For the purpose of evaluating the performance of data prediction, the prediction results are evaluated using root mean square error (RMSE), mean absolute error (MAE), and the coefficient of determination R2. The RMSE, MAE, and R2 can be computed by the following formulas:

27 $$\begin{aligned} \text {RMSE}= & \sqrt{\sum _{i=1}^n\left( Y_{pi}- Y_{ti}\right) ^2/ n} \end{aligned}$$

28 $$\begin{aligned} \text {MAE}= & \frac{1}{n}\sum _{i=1}^n\mid Y_{pi}-Y_{ti}\mid \end{aligned}$$

29 $$\begin{aligned} \text {R}^2= & 1-\frac{\sum _{i=1}^n(Y_{pi}-Y_{ti})^2/ n}{\sum _{i=1}^n(Y_{ti}-\overline{Y_t})^2/n} \end{aligned}$$

where *n* is the number of samples, $Y_{pi}$ is the *i*-th predicted value, $Y_{ti}$ is the *i*-th actual value, and $\overline{Y_t}$ is the average of the actual values. The closer $R^2$ is to 1, the better the prediction effect of the model is.

### Data prediction

In this paper, the BiLSTM neural network uses the sigmoid function as the activation function of neurons. A total of 122 days of data from 1st May 2023 to 30th August 2023 were used as the training set, data from 31st August 2023 were used as the test set, and the RMSE, MAE, and $\hbox {R}^{2}$ are used as the evaluation index of data prediction. The threshold *r* and the learning efficiency $\eta$ of the error gradient are 0.1 and 0.05, respectively, and the learning factors $dec_{r}$ and $dec_{\eta }$ are 0.05 and 0.8, respectively. The training set and the test set are inputted into the network after the completion of the training, and the prediction value $Y_{train}$ of the training set and the prediction value $Y_{a}$ of the test set are computed to obtain the training set’s predicted value and the residuals are calculated as $Y_{e}= Y_{a}-Y_{train}$.

**Fig. 7:**
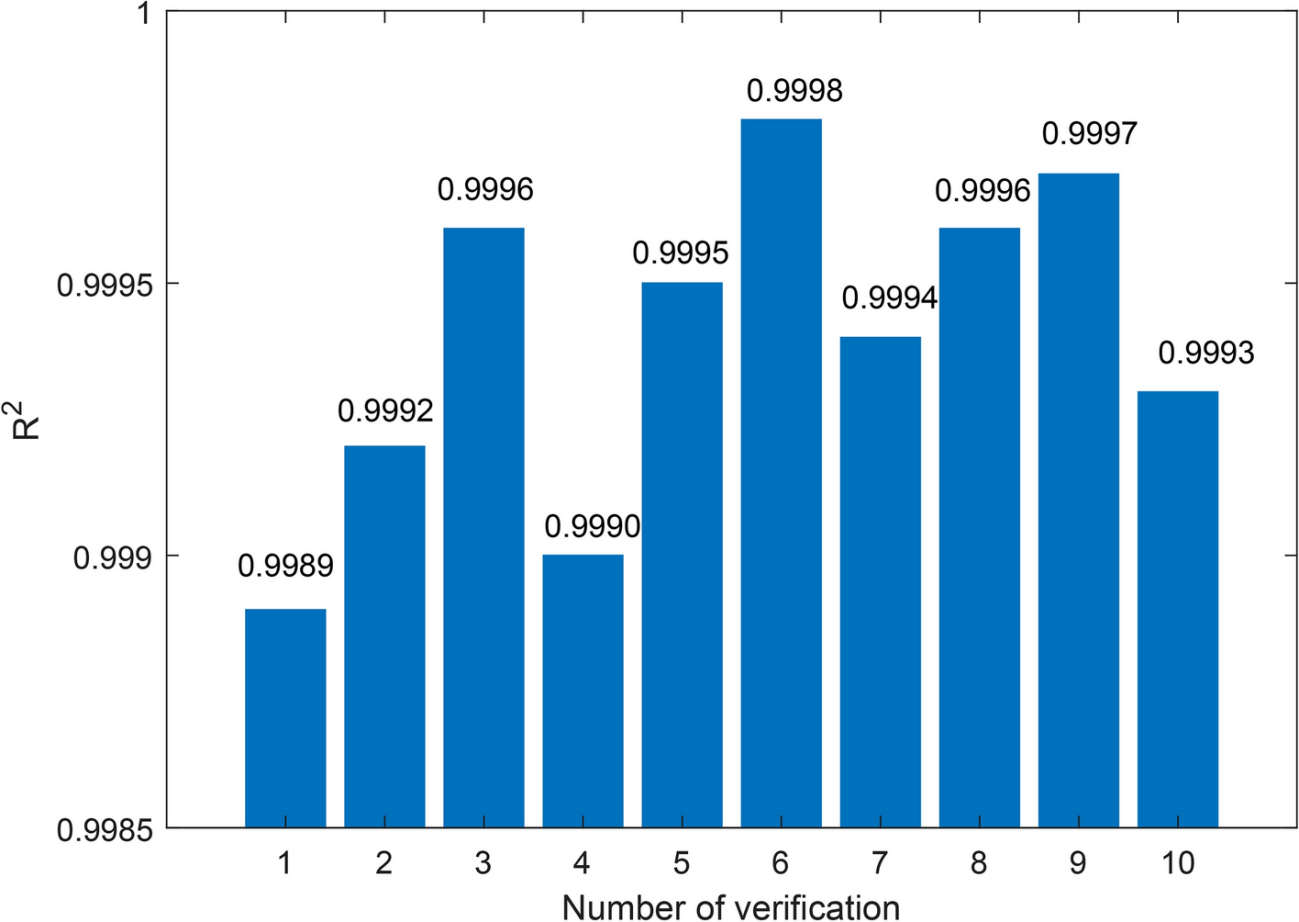
The result of the ten-fold cross-validation.

In order to verify the reliability and superiority of the CNN-BiLSTM-Attention model used in this paper on the prediction of the battery charge state of an electric loader, the data of winding temperature is selected for prediction analysis, and experimental comparisons are also made with support vector machine (SVM), CNN-LSTM and CNN-LSTM-Attention models. Using the ten-fold cross-validation method, the dataset is divided into ten subsets, nine of which are used as the training set and the remaining one as the validation set in turn, and the training is repeated ten times. The prediction results and errors of several models are shown in Table tab.Prediction errors of different models, and the ten-fold cross-validation results of the CNN-BiLSTM-Attention model are shown in Fig. 7. It can be seen that the model used in this paper has an accuracy of up to 0.9998 and down to 0.9989 in the ten-fold cross-validation experiments, with an average accuracy of 0.9994. It shows that the model does not fall into the overfitting state under different data subsets and has some stability. In addition, the average MAE of the model proposed in this paper is 0.474% and the average RMSE is 0.586%. Compared with the CNN-LSTM model, the MAE and RMSE are improved by 28.07% and 28.80%, respectively, and compared with the CNN-LSTM-Attention model, the MAE and RMSE are improved by 14.29 and 15.56%, respectively, which are superior to some extent.

**Table 1:** Prediction errors of different models.

Algorithm	MAE	RMSE	R^2^	Training time (s)
BP	2.721	23.073	0.9547	35
SVM	5.423	6.259	0.9612	47
LSTM	0.717	0.891	0.9785	51
CNN-LSTM	0.659	0.823	0.9871	75
CNN-LSTM-Attention	0.553	0.694	0.9986	88
CNN-BiLSTM-Attention	0.474	0.586	0.9994	102

Combined with Fig. 7 and Table 1, it can be found that the CNN-BiLSTM-Attention model is significantly better than the CNN-LSTM-Attention model. This is because the BiLSTM structure can read the data information in the forward and backward direction respectively, mine the intrinsic connection between the data, fit the current data, and improve the prediction accuracy. Hence, the proposed algorithm brings together the advantages of multiple models and results in the highest accuracy, while at the same time the computational speed is relatively slow. In summary, the CNN-BiLSTM-Attention model used in this paper can predict the winding temperature data more accurately and has a certain generalization ability. Meanwhile, the CNN-BiLSTM-Attention prediction model is applied to the composition of dissolved gas, upper oil temperature, and winding temperature in this paper.

### Intelligent fault diagnosis

Upper oil temperature and winding temperature can directly reflect the oil overheating and winding overheating in the transformer. The composition of dissolved gases in the oil is a condition assessment index reflecting the specific faults inside the transformer. The transformer is in normal operation, and the dissolved gases in the oil mainly include O_2_ and N_2_. When the transformer fault occurs, the composition and concentration of dissolved gases in the oil will change, and the characteristic gases of the fault may include $\hbox {H}_{2}$, $\hbox {CH}_{4}$, $\hbox {C}_{2}\hbox {H}_{2}$, $\hbox {C}_{2}\hbox {H}_{4}$, $\hbox {C}_{2}\hbox {H}_{6}$, $\hbox {CO}$ and $\hbox {CO}_{2}$. Different fault types correspond to different components of the characteristic gas. The main types of transformer faults and the corresponding gas composition are shown in Table 2, and the residual values of gas composition prediction and warning values are shown in Table 3.

**Table 2:** Fault type and corresponding gas composition.

Fault type	H_2_	CH_4_	C_2_H_2_	C_2_H_4_	C_2_H_6_	CO	CO_2_
Partial discharge	$\surd$	$\surd$	–	–	–	–	–
Arc discharge	$\surd$	$\surd$	$\surd$	$\surd$	–	–	–
Upper oil temperature 140 °C	–	$\surd$	$\surd$	$\surd$	$\surd$	–	–
Solid material	–	–	–	–	–	$\surd$	$\surd$

**Table 3:** The residual values of gas composition prediction and warning values.

Constituent	Prewarning value μL/L	Constituent	Prewarning value μL/L
$\hbox {H}_{2}$	25.20	$\hbox {C}_{2}\hbox {H}_{6}$	15.1
$\hbox {CH}_{4}$	11.40	$\hbox {CO}$	315
$\hbox {C}_{2}\hbox {H}_{2}$	1.55	$\hbox {CO}_{2}$	1850
$\hbox {C}_{2}\hbox {H}_{4}$	5.60		

**Fig. 8:**
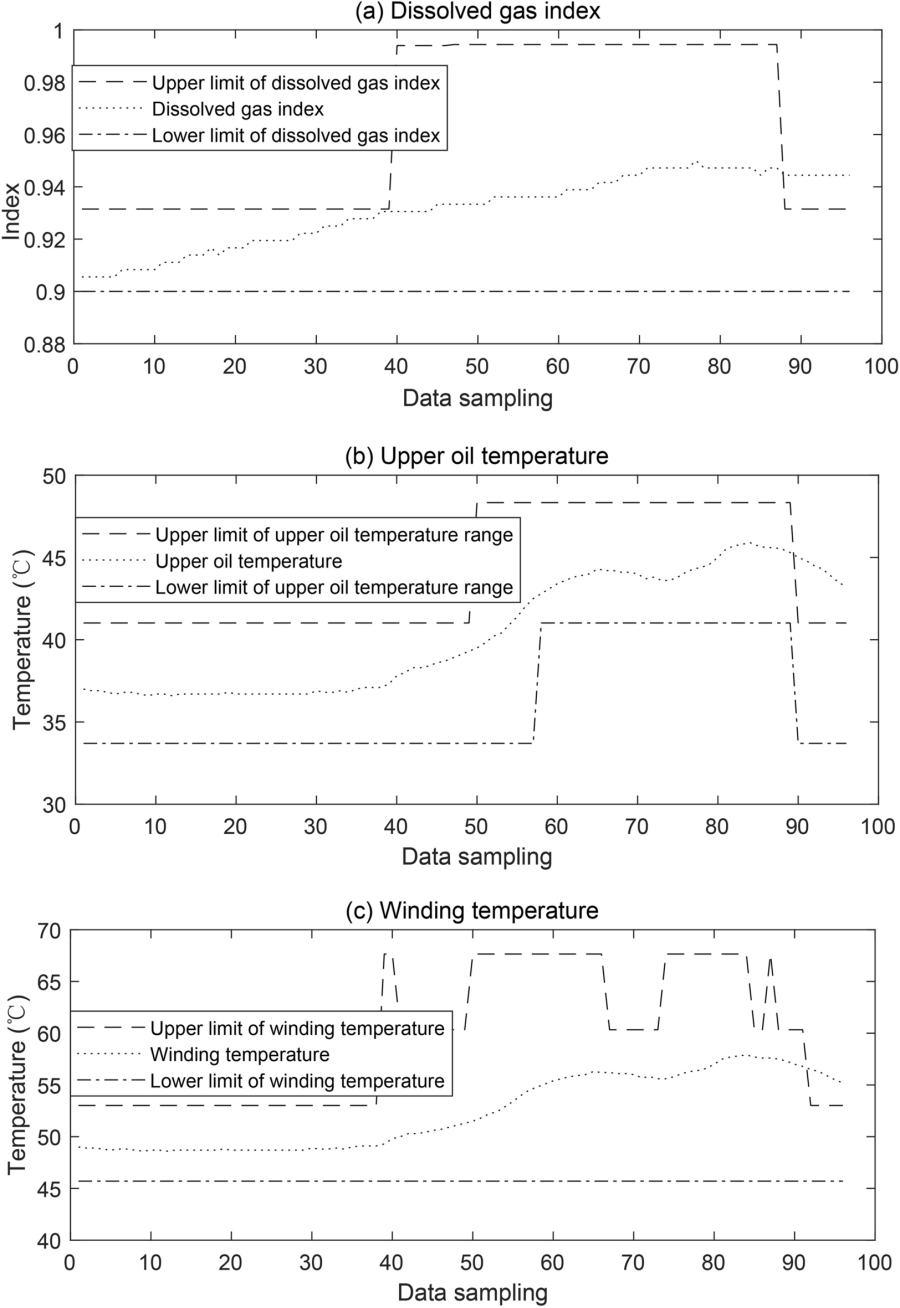
Intelligent fault diagnosis of transformer based on multi-source data fusion and correlation analysis.

Through the correlation analysis of the transformer load rate and the data of transformer oil dissolved gas, upper oil temperature, and winding temperature, the correspondence between the current load rate and the data of dissolved gas, upper oil temperature, and winding temperature is obtained, and six data correspondence intervals are divided at an environment temperature of 10 °C, as shown in Table 4. Based on the data in Table 4, the curves of dissolved gas, upper oil temperature, and winding temperature data are obtained under different loads, so as to carry out a short-term predictive assessment of the transformer. The three-phase winding temperature rise intervals and predicted temperature rises are shown in Fig. 8.

**Table 4:** Corresponding interval for correlation analysis under the different load rates.

Load rates	Dissolved gas index	Upper oil temperature (°C)	Winding temperature (°C)
$0 \le L \le 12.610$	$\left[ 0.805, 0.837 \right]$	$\left[ 33.700, 41.016 \right]$	$\left[ 45.700, 53.017 \right]$
$12.610 \le L \le 25.230$	$\left[ 0.805, 0.837 \right]$	$\left[ 33.700, 41.016 \right]$	$\left[ 45.700, 53.017 \right]$
$25.230 \le L \le 37.840$	$\left[ 0.837, 0.868 \right]$	$\left[ 41.016, 48.333 \right]$	$\left[ 45.700, 60.333 \right]$
$37.840 \le L \le 50.450$	$\left[ 0.868, 0.900 \right]$	$\left[ 41.016, 48.333 \right]$	$\left[ 45.700, 67.651 \right]$
$50.450 \le L \le 63.070$	$\left[ 0.900, 0.963 \right]$	$\left[ 41.016, 70.283 \right]$	$\left[ 45.700, 67.651 \right]$
$63.070 \le L \le 75.680$	$\left[ 0.900, 0.994 \right]$	$\left[ 48.333, 77.600 \right]$	$\left[ 53.017, 67.651 \right]$

As can be seen from Fig. 8, in the vicinity of sample point 88, the dissolved gas, upper oil temperature, and winding temperature data are not within the normal range of intervals and have not returned to the normal range until sample point 96, then it indicates that there may be a fault within the transformer. At the same time, through the detection of gas composition found that $\hbox {C}_{2}\hbox {H}_{2}$ and $\hbox {C}_{2}\hbox {H}_{4}$ exceeded the warning value, and it is presumed that the arc discharge phenomenon. The power monitoring platform sends out an early warning message and issues a notification for timely overhaul and maintenance. In addition, the cause of the transformer fault was determined by realizing the field test on site, and the fault type was the same as that detected by the method proposed in this paper. Through the field test to confirm the cause of the discharge and oil filtration, degassing treatment processing, to achieve the prediction of the accident early warning, to prevent further expansion of the accident.

From the analyses in Table 4 and Fig. 8, it can be seen that the composition of dissolved gases, the upper oil temperature, and the winding temperature of the transformer are affected at different load rates. In addition to this, the stable operation of the transformer is also affected by the environment temperature. The standard regulations for oil-immersed transformer oil in direct contact with the atmosphere of the top oil temperature rise shall not exceed 55 °C, and the average temperature rise of the windings shall not exceed 65 °C. The transformer will often work at 80–1000 °C, long-term in the role of higher temperatures will gradually age brittle, in the range of 80–140 °C, the transformer temperature rises 8 °C for each, and the shortening of its insulation life of about half. Normal environment amorphous alloy transformer is in rated operating conditions, the temperature rise will not exceed the limit value, but due to the weather is too cold or hot, will cause the transformer to run in harsh environments, so that the oil-immersed self-cooling transformer’s cooling capacity is weakened, the cooling effect is reduced. Therefore, when the amorphous alloy transformer operates in harsh environmental conditions, the temperature rise of the transformer’s high and low-voltage windings should be very careful.

In order to verify the validity of the model under different operating conditions of the transformer, the amorphous alloy transformer temperature field under different environment temperatures is also studied, respectively, to analyze the corresponding interval for correlation analysis under − 10 °C, 0 °C, 10 °C, 20 °C, 30 °C, 40 °C environment temperatures at 50% load rate in Table 5.

**Table 5:** Corresponding interval for correlation analysis under the different environment temperatures.

Environment temperatures (°C)	Dissolved gas index	Upper oil temperature (°C)	Winding temperature (°C)
− 10	$\left[ 0.356, 0.386 \right]$	$\left[ 29.856, 30.003 \right]$	$\left[ 39.310, 39.635 \right]$
0	$\left[ 0.508, 0.518 \right]$	$\left[ 39.017, 39.255 \right]$	$\left[ 39.268, 39.473 \right]$
10	$\left[ 0.627, 0.634 \right]$	$\left[ 48.135, 48.266 \right]$	$\left[ 50.364, 50.471 \right]$
20	$\left[ 0.756, 0.772 \right]$	$\left[ 59.024, 59.318 \right]$	$\left[ 61.087, 61.352 \right]$
30	$\left[ 0.883, 0.904 \right]$	$\left[ 71.267, 73.586 \right]$	$\left[ 83.281, 85.636 \right]$
40	$\left[ 0.991, 0.996 \right]$	$\left[ 95.558, 98.215 \right]$	$\left[ 101.0386, 103.659 \right]$

**Fig. 9:**
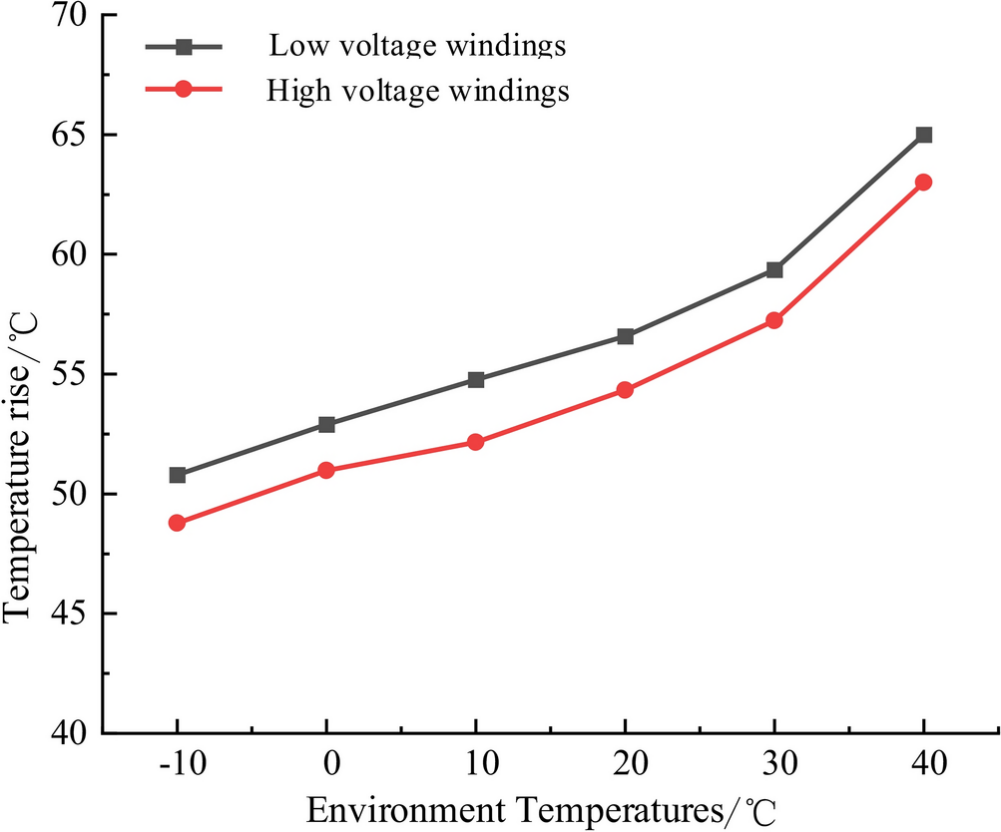
The temperature rise of high and low voltage transformer winding under different environment temperatures.

Additionally, this section also analyzes the temperature field distribution of high and low-voltage windings under − 10 °C, 0 °C, 10 °C, 20 °C, 30 °C, 40 °C environment temperatures. Figure 9 gives the temperature rise of high and low voltage transformer winding under different environment temperatures, as can be seen from the figure, the environment temperature within 35 °C, the high and low voltage winding temperature rise rises gently and does not exceed 65 °C temperature rise limit. When the environment temperature is more than 35 °C, the slope of the curve increases sharply, the high and low-voltage winding temperature rise increases markedly and has even exceeded the 65 °C limit value of the temperature rise, the normal operation of the transformer and winding insulation pose a great threat to the normal operation of the transformer, so the transformer is easy to be damaged when running in a high-temperature environment, especially when the environment temperature exceeds 35 °C.

### Comparative analysis of fault diagnosis for multiple models

Five mainstream supervised learning models, namely, Linear Discriminant Analysis (LDA), K-Nearest Neighbour Algorithm (KNN), SVM, Random Forest (RF), and gradient boosting decision tree (GBDT), are selected to be trained under empirical parameters, and the test results are compared with the proposed model, and the results are shown in Table 6. As shown in the table, the diagnosis accuracy of the proposed model is the highest among the six models.

**Table 6:** Accuracy rate of fault diagnosis for different models.

Fault type	LDA	KNN	SVM	RF	GBDT	The proposed method
Partial discharge	0.692	0.818	0.753	0.955	0.923	0.936
Arc discharge	0.909	1	0.692	0.918	0.906	1
Upper oil temperature 140 °C	0.923	0.875	0.894	0.923	0.885	1
Solid material	0.768	0.471	0.692	1	1	1

In order to demonstrate and illustrate the computational stability of the proposed method in this paper, 100 consecutive random samples of the sample set are performed, each time 20% of the samples are taken as the test set, and the remaining 80% of the samples are taken as the training set. The obtained training samples are used to train different models and the diagnosis results are counted and the results are shown in Table 7. As can be seen from the table, the average correct fault diagnosis rate of 100 diagnoses of the transformer fault diagnosis model proposed in this paper is 0.917, and the mean square error of the correct rate is 0.018. Compared with five mainstream supervised learning models, the transformer fault diagnosis model proposed in this paper has the highest correct rate, and the mean square error of the correct rate is smaller compared with the RF and GBDT models which have higher accuracy, which indicates that the method proposed in this paper is able to stably maintain the computational output with high accuracy.

**Table 7:** Diagnosis results of different models with repeated training.

Methods	Highest correct rate	Lowest correct rate	Mean correct rate	Mean variance of correct rate
LDA	0.709	0.518	0.611	0.038
KNN	0.845	0.706	0.741	0.044
SVM	0.802	0.718	0.724	0.035
RF	0.936	0.855	0.873	0.032
GBDT	0.945	0.818	0.887	0.026
The proposed method	0.982	0.891	0.917	0.018

## Conclusions

In this paper, a novel intelligent fault diagnosis of transformers based on multi-source data fusion and data mining has been developed to figure out issues of faults and realize the prognostics and health management under multiple operation conditions in the power transformer. Above all, an improved entropy weighting method is employed to achieve the data fusion of various components for transformer dissolved gases. Then, the load rate, upper oil temperature, winding temperature data, and the fusion indices of dissolved gas components in the transformer are predicted by the combination of a bidirectional long short-term memory network, attention mechanism, and convolution neural network. In addition, for the purpose of the predictive assessment of the transformer state, Apriori correlation analysis based on the support and confidence levels, is performed on the transformer load rate, upper oil layer, winding temperature, and fusion indices of gas components. The specific conclusions to be drawn are the following:

1. Aiming at the problems of transformer operating conditions and loads, complicated parameters, and difficulty in effectively achieving the state predictive assessment, the proposed method is the method based on the data prediction and correlation analysis method to assess the health state of the transformer. Compared with past research, the proposed method can include a variety of characteristic parameters. Effective matching and correlation analysis of the characteristic parameters under different load rates of the predicted data is based on real-time assessment to improve early warning capability. The results show that in the vicinity of sample point 88, the dissolved gas, upper oil temperature, and winding temperature data are not within the normal range of intervals, and it is presumed that the arc discharge phenomenon.
2. By using the method of multi-source data fusion and data mining, the operating state of the transformer can be preliminarily judged by the data change of upper oil temperature, winding temperature data, and the fusion indices of dissolved gases components, which provides a simple and efficient intelligent online monitoring method for transformers that have been put into use, and also an effective method to identify a single phase fault. The experiment result shows that the transformer is easy to be damaged when running in a high-temperature environment, especially when the environment temperature exceeds 35 °C.
3. Compared with the method of setting thresholds, the method proposed in this paper can sense the operating situation of the equipment in advance and take corresponding measures to reduce the incidence of accidents and improve the reliability of the power supply. Compared with five learning models, i.e., LDA, KNN, SVM, RF, and GBDT, the transformer fault diagnosis model proposed has the highest correct rate, and the mean square error of the correct rate is smaller. The average correct fault diagnosis rate of 100 diagnoses of the transformer fault diagnosis model proposed in this paper is 0.917, and the mean square error of the correct rate is 0.018.

## Data Availability

The datasets generated during and/or analyzed during the current study are available from the corresponding author (Jingping Cui) on reasonable request.
